# Evolutionary medical insights into the SARS-CoV-2 pandemic

**DOI:** 10.1093/emph/eoaa036

**Published:** 2020-10-14

**Authors:** Bernard Crespi

**Affiliations:** Department of Biological Sciences, Simon Fraser University, 8888 University Drive, Burnaby, BC V5A 1S6, Canada

**Keywords:** COVID-19, SARS-CoV-2, inflammation, interferon, coronavirus, amlodipine

## Abstract

The author apply concepts and tools from evolutionary medicine to understanding the SARS-CoV-2 pandemic. The pandemic represents a mismatched conflict, with dynamics and pathology apparently driven by three main factors: (i) bat immune systems that rely on low inflammation but high efficacy of interferon-based defenses; (ii) viral tactics that differentially target the human interferon system, leading to substantial asymptomatic and pre-symptomatic transmission; and (ii) high mortality caused by hyper-inflammatory and hyper-coagulatory phenotypes, that represent dysregulated tradeoffs whereby collateral immune-induced damage becomes systemic and severe. This framework can explain the association of mortality with age (which involves immune life-history shifts towards higher inflammation and coagulation and reduced adaptive immunity), and sex (since males senesce faster than females). Genetic-risk factors for COVID-19 mortality can be shown, from a phenome-wide association analysis of the relevant SNPs, to be associated with inflammation and coagulation; the phenome-wide association study also provides evidence, consistent with several previous studies, that the calcium channel blocking drug amlodipine mediates risk of mortality.

**Lay Summary**: SARS-CoV-2 is a bat virus that jumped into humans. The virus is adapted to bat immune systems, where it evolved to suppress the immune defenses (interferons) that mammals use to tell that they are infected. In humans, the virus can apparently spread effectively in the body with a delay in the production of symptoms and the initiation of immune responses. This delay may then promote overactive immune responses, when the virus is detected, that damage the body as a side effect. Older people are more vulnerable to the virus because they are less adapted to novel infectious agents, and invest less in immune defense, compared to younger people. Genes that increase risk of mortality from SARS-CoV-2 are functionally associated with a drug called amlodipine, which may represent a useful treatment.

## INTRODUCTION

The SARS-CoV-2 pandemic is an ongoing evolutionary process. A bat-adapted virus has undergone a host shift into humans, where it has been subject to novel selective pressures. In turn, humans are being subjected to a novel infectious agent to which they are not well adapted. Thus far, responses to the virus have centered primarily on public-health measures, searches for treatments based on existing antiviral therapies and comparable human symptoms, and the development of vaccines. How can evolutionary-medical approaches help?

The purpose of this Commentary is to apply the fundamentals of evolutionary medicine to understanding the ecology, evolutionary biology and epidemiology of the SARS-CoV-2 virus, and to treatment or prevention of the associated disease COVID-19. The main goal is to develop and evaluate a robust hypothesis for the adaptive significance of the primary phenotypes of the virus, in relation to its pathological effects on humans. To reach this goal, I draw on five primary lines of evidence: (i) bat life histories and behavior, (ii) bat immune systems, (iii) human immune systems, (iv) coronavirus life histories and adaptations and (v) COVID-19 symptoms, treatments, epidemiology and preventatives. The main criterion for robustness of the hypothesis is convergence of evidence from across these domains. In this context, the role of evolutionary thinking and approaches is to help direct researchers along novel and promising paths, by developing potential explanations and predictions that may not otherwise be discerned.

### Bat life histories and behavior

Bats, like humans, represent outstanding hosts for viruses because they live in large, dense populations (facilitating spread of pathogens), engage in air travel (that can spread virus between populations), live in enclosed, protected habitats (that are amenable to virus survival), exhibit high longevity (that can favor long-term viral persistence in a given host) and vocalize (that can propel viruses to new hosts) [1]. Whether or not bats harbor more viruses than expected for a taxon of their size, the viruses that they do host are clearly much more virulent, upon transfer to non-bat species including humans, than are viruses from other mammals [2]. In contrast, for the bats themselves, such viruses appear to typically exhibit minimal impacts on health [1]. A simple explanation for low virulence in bats, but high virulence following host-shifts to non-bats, is that bats have been subjected to exceptionally strong selection from viruses throughout their evolutionary histories, for the ecological and behavioral reasons just described. Viruses have likewise exhibited strong selection upon humans [3], but apparently for much shorter periods of evolutionary time, on the order of tens of thousands of years at high host densities, compared with tens of millions for bats.

Strong selection on bats from viruses is evidenced most strikingly by the divergences of bats from other mammals in major features of their immune systems [4], and by extensive evidence for strong positive selection on bat immune genes, including at the origin of the Chiroptera [5]. The specific nature of such divergences is key to understanding the strategies and tactics used by bats to resist viral pathogens, in comparison to those used by humans.

### Bat immune systems

The immune systems of bats differ from those of other mammals in several fundamental ways. First, bats use inflammation as an anti-pathogen defense to a much lesser degree [4, 6, 7]. This divergence has been attributed to the unique bat adaptation of flight, which involves high metabolic rate, elevated body temperature, and consequent increases in reactive oxygen species production [6]; these circumstances are surmised to have led to the evolution of novel anti-inflammatory mechanisms that are also expressed in the context of immunity, with additional benefits from reduction in collateral tissue damage from pro-inflammatory immune responses [8, 9]. This hypothesis can also help to explain the strikingly long lifespans of bats for their body sizes, given the central roles of inflammation in aging [10, 11].

In addition to these broad differences between the immune systems of bats and other mammals, it is important to note that bats are a large and highly diverse group that exhibits considerable variation among species in the details of their immune adaptations.

Reduced inflammatory responses in bats, compared with other mammals, create a notably different immune environment for viruses, where they may more easily persist at relatively low and less harmful levels [4, 12]. In this context, bat viruses are also less subject to selection from inflammatory responses (than are non-bat viruses), and so evolve their host-related adaptations in response to selection from alternative bat antiviral defenses. Similar considerations probably also apply to birds, with implications for human avian-derived influenza, but inflammation in birds has been subject to little study, and mainly in a model system that is, unfortunately, nearly unable to fly (chickens).

The second major immune-system difference between bats and other mammals is that bats exhibit notable expansions and specializations in their deployment of anti-viral interferons [13, 14]. Interferons, especially those of so-called Type 1, represent the primary line of vertebrate immune defense that is specific to viruses [15]. Most broadly, interferons activate local and systemic cytokine responses, trigger acute-phase responses and sickness behavior, and initiate the adaptive immune responses that can lead to the eventual production of antibodies [16, 17]. Interferon-related adaptations in bats include, e.g. constitutive and highly inducible expression, and expanded and divergent ranges of interferon-induced genes [18]. More generally, arms races of host interferon systems with viral anti-interferon tactics, in bats and other mammals, represent some of most diverse and complex molecular–conflictual interactions yet described, that lead to diverse outcomes in both bats and viruses that are expected to be specific to each host-virus interaction [19–21]. Highly effective interferon-centered defenses can help to keep viruses at low levels and tolerated, rather than having them be driven by selection to immediately replicate to high densities, to facilitate transmission to new hosts before being destroyed by a high-intensity immune response [12, 22, 23].

The upshot of these considerations is that bat immune systems are characterized by enhanced deployment of interferon-based defenses, and reduced use of defenses based on inflammation. Both of these immune differences from other mammals appear to contribute to bat tolerance of viruses, coupled with low virulence in the natural host, at least until host resistance declines due to advanced age or stress, when especially rapid replication may ensue [24, 25]. As a corollary, bat viruses are expected to have been subject to especially strong selection for subversion of interferon systems, with important implications in the event of shifts to non-bat hosts. Understanding how bats limit pathological effects from coronaviruses in general, and SARS-CoV-2 in particular, should be a high priority for research on COVID-19, with special focus on how the immune systems of horseshoe bats, the apparent natural hosts of SARS-CoV-2, interact with this virus. An important evolutionary insight in this situation is the intensity, diversity and species specificity of antagonism and defense between viruses and bats, and just how molecular–conflictual interactions are perturbed upon transfer of a bat virus to a new, non-bat host.

### Human immune systems

Bat viruses in a human body represent a ‘double’ evolutionary mismatch, with both species subject to novel effects and environments to which they are not adapted. The specific outcomes of such mismatches are idiosyncratic and unpredictable *a priori*, and depend on the details of the disparities: on one hand, the virus has escaped the specialized, evolved defenses of its natural bat host, but on the other hand, it is faced with a divergent cellular and molecular human host landscape, especially with regard to receptor distributions and immune responses. Mismatches subsequent to host shifts from bats to humans, and perhaps including an intermediate host as well, thus generate a situation where the clinical effects of a virus in its new host can be challenging to predict, although they should depend to some extent on its adaptations in the ancestral host.

Unlike bats, humans rely heavily on inflammation as a quickly acting, non-specific defense against infection or injury; this system has been designed by selection to work locally at the impacted site, via orchestration of short-term cellular responses that destroy pathogens but can also be subject to tradeoffs: damage to one’s own tissues, especially if the infection becomes widespread or prolonged, or anti-inflammatory responses are slow to restore equilibrium [8, 26]. Such ‘double-edged’ inflammatory responses work hand in hand, and synergistically, with the coagulation and complement systems [27, 28], with coagulation serving to help spatially restrict the infection and seal-damaged blood vessels, but also raising the risk of damaging localized, or more systemic, thrombosis (clotting and ischemia). Inflammation and coagulation are thus not pathological, but are subject to actual or potential costs (especially if dysregulated or systemic, in some individuals), as well as providing clear and substantial benefits.

Bat viruses are predicted to not be well adapted to host inflammatory responses, which are reduced in their hosts. But they are, as described above, specialized at subversion of interferons, and most importantly, interferons are upstream of inflammatory cascades: without interferon-based signaling, the body’s brain (cytokines and the immune system), and the brain itself (the acute-phase responses and sickness behavior), will simply be unaware that an infection is taking place. Until, perhaps, it is too late for this type of response to be appropriate or effective.

### Coronavirus life histories and adaptations

The considerations described earlier regarding bat and human immune systems motivate the hypothesis that interactions between humans and bat-derived respiratory viruses are mediated by mismatches (especially with regard to the interferon and inflammation–coagulation systems) that have imbalanced the dynamics of conflicts between viruses and hosts. By this hypothesis, infection is characterized by strong initial suppression of host defenses and delay of human interferon-based response, accompanied by a rapid increase in viral load. In vulnerable individuals, activation of inflammatory and coagulatory responses then occurs too late for local effectiveness, and too strongly (due to the high, widespread viral load), such that tissue damage becomes widespread and severe. Such high virulence is expected to be maladaptive for the virus (as well as for the host), since viral transmission is reduced by host immobilization and death. This hypothesis fits well with coronavirus-induced pathology in general and COVID-19 pathology in particular [29–31], and is evaluated in more detail below. Under the hypothesis, the SARS-CoV-2 virus has evolved especially effective anti-interferon mechanisms (better than those in other viruses, many of which also antagonize interferons), that mediate its high virulence under mismatched, human-host conditions.

For interactions driven by mismatches and conflicts, the devils are in the details, here represented by the genomes, proteins and life history strategies of coronaviruses and SARS-CoV-2. Coronaviruses infect the respiratory or gastrointestinal tracts of mammals and birds. They are unusual among RNA viruses in the large sizes of their genomes (about 25–30 kb); most RNA viruses have poor or absent RNA repair, and so are restricted to small genome sizes lest they cross the ‘error threshold’ of too many highly deleterious mutations incurred during replication [32]. Coronaviruses have, in contrast, evolved their own system of repair that allows relatively faithful replication [33, 34]. A large genome, for a virus, means more genes, which means more weaponry for exploiting the host. SARS-CoV-1, e.g. which is very similar genetically and phenotypically to SARS-CoV-2, harbors at least eight genes that antagonize interferons, within a larger suite of at least ten genes that modulate innate immunity [13], and (MERS) exhibits a comparable suite of anti-interferon genes for immune-system evasion [35]. Middle East Respiratory Syndrome virus.

Of the seven coronavirus species that infect, or have infected humans, three are recent zoonotics (SARS-CoV-1 and -2 and MERS) that cause relatively severe disease in vulnerable hosts, and four (229E, NL63, HKU1 and OC43) are long-established zoonotics that cause mild disease (‘common colds’), although they have the capacity to cause severe or fatal lung disease in the frail or elderly [36]. SARS-CoV-1 and MERS appear to spread poorly between humans in non-medical settings, and not pre-symptomatically [37] at least in part due to their high virulence, whereas 229E, NL63, HKU1 and OC43 spread quite effectively, as evidence by high seropositivity in children [38, 39]; these viruses can also exhibit notable rates of asymptomatic or weakly symptomatic infection [40]. In the context of phenotypes of other human coronaviruses, SARS-CoV-2 thus appears to combine the asymptomatic or mildly symptomatic (and, presumably, pre-symptomatic) spread of 229E, NL63, HKU1 and OC43 with the high virulence of SARS-CoV-1 and MERS.

High virulence (morbidity and mortality) is unexpected in directly transmitted pathogens, from basic evolutionary-medical theory, because it normally engenders reduced mobility of the host [41]. Thus, directly transmitted viruses that are highly virulent are selected against because they reduce the mobility of their hosts, or kill them, in both cases reducing their own opportunities to transmit. A key assumption of this paradigm is that virulence and transmission rates trade off, due to a necessary association of substantial viral load with both high virulence and low mobility. SARS-CoV-2 appears to at least partially break this tradeoff, because it is commonly transmitted by people who are non-symptomatic or pre-symptomatic and thus mobile, and because high virulence develops only later in the infection process, and only in some individuals.

SARS-CoV-2 virulence, and transmission dynamics, are especially interesting in the context of how the application of public health measures and therapeutics are predicted to impact evolution of the virus [42]. First, effective quarantine of symptomatic individuals should select for a longer pre-symptomatic period (and viral genotypes that produce asymptomatic cases), because quarantine causes death for the viruses in that host. Such selection should, moreover, not favor the evolution of lower or higher virulence, since all quarantined viruses are dead in any case. In contrast, if individuals with less virulent strains of the virus are less likely to be ascertained and quarantined, then selection should favor reduced virulence.

Second, selection always favors viral genotypes with a higher transmission rate, with a greater strength of such selection in more-dense populations [42]; this effect may be represented by the 614G allele in SARS-CoV-2, which has increased very rapidly and shows functional differences in its spike protein from the ancestral allele D614 [43, 44]. Social distancing, which reduces effective host density, is predicted to weaken the strength of selection for higher transmission rates (though extend the duration of selection overall), likewise with no expected effect on virulence [42]. The main way that quarantine, or social distancing, could affect virulence under these scenarios would be indirectly, via pleiotropic effects of genotypes that code, presumably, for longer pre-symptomatic periods or more-effective transmission [42]. Such effects depend upon details of mechanisms; e.g. does a longer pre-symptomatic period involve better host interferon-system suppression, leading later to higher viral loads and increased virulence? Does enhanced transmission lead to increased initial doses of virus? The main implication of these predictions is that this type of virus is not necessarily expected to evolve reduced virulence as an effect or means of increasing its transmission. This expectation is reinforced by the observation that virulence of SARS-CoV-2 appears to be less a function of the direct impacts of the virus, and more a consequence of innate immune-system hyper-reactivity, in vulnerable subsets of the population, that follows from the mismatches between virus attack and host defense.

Third, therapeutic agents are expected to impose strong selection on the virus, leading to the evolution of drug-resistant genotypes. The degree to which such resistant genotypes will arise and spread outside of health-care contexts will depend on how commonly the therapeutic agent is used (with increased application leading to faster resistance evolution), the costs of resistance (i.e. relevant tradeoffs for the virus), and whether the virus can be effectively cleared by the therapy plus the immune response. ‘Evolution-proof’ therapies can be developed, in theory, to pre-empt resistance [45]; for coronaviruses, such therapies might target their unique RNA-replication repair system. If sufficiently effective, this strategy could drive the virus over the error threshold to mutational meltdown because every new genome would harbor deadly or strongly deleterious mutations.

### COVID-19 symptoms, treatments, epidemiology and preventatives

The diversity of COVID-19 symptoms can, in principle, be broadly interpreted in the context of coronavirus adaptations and differences between bat and human hosts. Thus, high rates of asymptomatic and pre-symptomatic transmission [46, 47] appear to reflect especially effective interferon-system suppression by the virus, the widespread multi-organ symptomatology reflects the human, compared with bat, tissue distribution of ACE2 receptors, and immune hyper-reactions appear to reflect the human reliance on inflammatory and anticoagulation defense systems, here in inappropriate and maladaptive contexts [48]. Bats, in contrast, are typically asymptomatic (like some humans) and tolerate the virus, normally being able to keep it at low enough levels to avoid pathological effects. This hypothesis can by no means fully or solely account for all or many of the clinical features and details of COVID-19 infection, but it provides a general starting point, and guideline, for interpreting them in terms of mismatches, conflicts, coronavirus adaptations and immune differences between humans and bats that can help to guide data collection and clinical interventions.

A primary consequence of these considerations, as regards the causes and treatment of COVID-19, is that it should, as noted by some clinical researchers [31], be two-phased: (i) early treatment with anti-viral agents to suppress viral load, using agents to target viral replication and reduce or alleviate its suppression of interferon-based defenses, and (ii) later treatment, if needed, to modulate the pro-inflammatory and coagulation-defense arms of innate immunity (e.g. with dexamethasone) [49, 50], to prevent the hyper-reactions that follow from virus-host mismatches. Whether anti-viral treatments can be given early enough in the disease course to be very effective remains unclear, given the relatively long period of time that people are pre-symptomatic, and treatment with interferons need not be the best approach, because the virus may block interferon-related pathways at any number of points. Determining how horseshoe bats use interferons to control SARS-CoV-2, and how the virus has evolved to antagonize interferon pathways, become crucially important in this context.

The hypothesis that excessive immune-defense reactions involving inflammation and coagulation represent a primary cause of death from COVID-19 can be evaluated further, in a non-clinical manner, by determining if the main genetic risk factors for mortality from this disease are associated with these two immune-system domains. Seven SNPs have been associated thus far with COVID-19 mortality versus survival after infection [51, 52]. A phenome-wide association study (PheWAS) conducted by the author using the GWAS Atlas (https://atlas.ctglab.nl) that identifies phenotypes with which these SNPs have been associated from previous GWAS work, shows links of the SNPs with clotting, respiratory capacity, and aspects of innate immune cells, among many other traits (Table 1 and Supplementary Material).

**Table 1.** Phenome-wide analysis results for the SNPs associated with COVID-19 survival versus mortality^a^

SNP	Gene(s)	PheWAS phenotypes relevant to inflammation, coagulation and respiratory functions	Medication phenotypes that were reported for two or more SNP associations
rs657152	ABO blood group	Clotting time, monocyte count, HB concentration, (PEF) peak expiratory flow, CCL4	Amlodipine and aspirin
rs11385942	LZTFL1, CCR9 and others	Monocyte, granulocyte, neutrophil, macrophage and eosinophil traits; lymphocyte count, IL-18; IL-4, hypertension, antithrombotic agents, Type 2 diabetes, agents acting on renin-angiotensin system, blood clot, DVT (deep vein thrombosis), allergic and atopic diseases and BMI	
rs150892504	EVAP2	Platelet count and BMI	
rs138763430	BRF2	Lymphocyte count and FEV1/FVC ratio (forced expiratory volume/forced vital capacity)	Amlodipine
rs117665206	TMEM181	FEV1, PEF, monocyte chemotactic protein-1 (CCL2) and CCL4	Amlodipine
rs147149459	ALOXE3	FVC, PEF and FEV1	
rs151256885	ALOXE3 (intronic)	Blood clot, DVT, allergic and atopic diseases; eosinophil percentage	Amlodipine and aspirin

a These findings suggest that genetic risk of COVID-19 mortality is associated with blood clotting, various aspects of the innate immune system, respiratory capabilities, and effects of the drug amlodipine. The risk allele associated with the SNP rs11385942 was recently shown to have been derived from introgression of Neanderthal DNA into *Homo sapiens sapiens* [53]. Medications associated with a single SNP include atorvastatin, bendroflumethiazide, lansoprazole, metformin, paracetamol and simvastatin. CCL2 and CCL4 are chemokines implicated in ‘cytokine storms’ [48]. PheWAS phenotypes with GWAS *P*-values < 0.05 are included in the analyses. See Supplementary Material for full results. Replicated GWA studies of COVID-19 survival with large samples are needed for more robust determination of the full range of SNPs that mediate risk. The GWAS for use of amlodipine included 15 555 cases and 264 888 controls, and for use of aspirin there were 51 136 cases and 229 397 controls, with data from the UK Biobank [54]. The presence of multiple PheWAS associations for amlodipine, but not for hypertension, T2D or BMI (known COVID-19 mortality risk factors) for the same SNPs, suggests that some aspect or correlate of amlodipine treatment itself mediates mortality risk.

These results support roles for the associated SNPs with COVID-19 pathology, and more generally provide insights into the potential adaptive significance of the SNP variation in the immune, respiratory and vascular systems. Comparing PheWAS hits across the seven SNPs (Supplementary Material), the phenotype convergently associated with most of the SNPs (four) was ‘treatment/medication code: amlodipine’, meaning that these SNPs are associated with use of this drug (before and during the pandemic). Amlodipine is a calcium channel blocker, used to treat hypertension that also has anti-inflammatory and anti-coagulatory properties [55–57]. Two small, retrospective clinical studies [58, 59] have shown that use of amlodipine (and the related drug nifedipine, for one study) was associated with 3- to 4-fold reductions in COVID-19 mortality rates (26.1% vs 6.8%, and 50% vs 14.6%) among individuals with hypertension.

Amlodipine and several other calcium channel blocking drugs also substantially inhibit SARS-CoV-2 infectivity in an *in vitro* epithelial cell model system [60]. Prospective, double-blind, case-control studies could usefully test the efficacy of amlodipine more rigorously, and determine whether its apparent therapeutic mechanism includes dampening of excessive innate immune-system activity, among other effects such as interference with viral manipulation of calcium homeostasis in the host [61]. The only other drug associated with two or more SNPs in the PheWAS was aspirin (Table 1), which has well-known anti-inflammatory and anti-coagulant effects.

Signs, symptoms and severity of infectious disease can, in host-pathogen systems at some evolved equilibrium, be interpreted in terms of adaptations of the host, the pathogen or neither [62]. For mismatched systems like humans and SARS-CoV-2, ‘neither’ becomes a highly viable alternative; indeed, in severe COVID-19 the main cause of pathology and death is, by the hypothesis addressed here, the immune system rather than the virus. The question then becomes, what explains the main epidemiological correlates of mortality, especially increased age and male sex [63, 64], and how might this information help to guide treatment or prevention?

What is notably curious about COVID-19 disease is its wide spectrum of symptoms and virulence, from a lack or minimum of symptoms in many or most children, to high death rates in the elderly and in persons with pre-existing conditions such as obesity or diabetes. This overall pattern of increasing mortality with advanced age, and low virulence in children is not unusual among respiratory diseases [65, 66]. The causes of such low virulence in children remain unclear, but probably include the fact that children are adapted for exposure to novel viruses (and have high numbers of naive T cells), exhibit low rates of inflammatory conditions compared with the middle-aged or elderly, and, if pre-pubertal, are not subject to tradeoffs of reproduction with immunity and other aspects of maintenance [65, 66].

**Figure 1.**
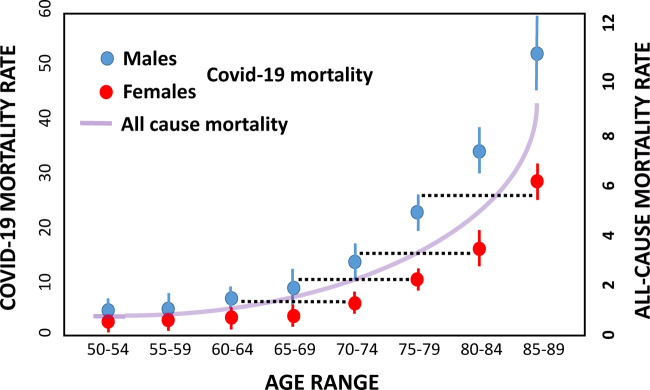
The mortality rate of females from Covid-19 is similar to the mortality rate of males who are about 10 years younger, chronologically, as shown by the dotted lines (data from Italy [69].). Given that males senesce and die, from all causes, about 8 years earlier than females, these data suggest that the higher male than female mortality from Covid-19 may be attributable mainly to the effects of biological age. The purple curve represents all-cause mortality for males and females combined (data from 2009 US death rates, CDC/NCHS, nchs/products/databriefs/db26.htm. The match in shape of this all-cause mortality curve to the Covid-19 mortality data suggest that biological factors associated with age are the primary cause of Covid-19 induced death.

Age is overwhelmingly the main risk factor for COVID-19 mortality, but male sex also shows strong effects, on the order of 1.5- to 2-fold for a given age class of older individuals [64]. The inference that these latter data indicate impacts of sex *per se* assumes, however, that males and females of similar chronological age are of similar biological age, with respect to factors increasing risk of death from COVID-19. This assumption is unwarranted. For well-established evolutionary reasons [67], males senesce and die earlier than females, with a typical difference of about 8 years overall [68]. At advanced ages relevant to senescence (over about age 60), if death is a function of biological age, then male mortality rates are expected to approximately match the mortality rates of females who are about 8 years older. This is essentially what we see in COVID-19, at least in the population analyzed here (Fig. 1). The hypothesis that case fatality rate age distributions of COVID-19 are direct consequences of biological age is also supported by the close match of the human all-cause mortality distribution with the distribution generated by COVID-19 (Fig. 1), and by ability to predict COVID-19 severity from biological markers associated with aging [70]. These considerations do not deny effects of male sex on mortality risk, but instead point out a single, more parsimonious explanation (biological age) for a substantial proportion of the risk currently attributed to differences between the sexes.

What specific aspects, then, of advancing age, are expected to increase risk of mortality from COVID-19? By the hypothesis described earlier, older individuals are expected to exhibit greater reliance on pro-inflammatory immune defense mechanisms, increased coagulatory responses, and reduced effectiveness of the adaptive immune system, including the generation of antibodies. Each of these three differences has been reported in the literature [71, 72], especially in the contexts of ‘inflammaging’ and immunosenescence [73, 74]. Obesity and Type 2 diabetes, which are among the two primary COVID-19 mortality risk factors in addition to age [75, 76], are also characterized by chronic inflammation [ 69, 76, 77].

Reduced efficiency of the adaptive immune system with age is expected from first principles of immune life history: antibody-based immunity represents, in large part, an investment in protection from future, recurrent pathogens, which become much less a factor with increasing age [9, 78]. What these considerations also mean is that the persons most in need of vaccine-based protection against SARS-CoV-2 infection will be least able to generate the required antibodies, as vaccination against nearly all pathogens becomes much less effective with age [79, 80].

For most individuals, and indirectly for the elderly, the best defense against SARS-CoV-2 will be vaccination. Vaccines, like antibiotics, can impose selection upon viruses. In particular, vaccines that are ‘imperfect’, in that they reduce morbidity and mortality but also allow for some degree of transmission, can allow the evolution and maintenance of higher virulence, because in such circumstances viruses are protected from the immobilizing or killing of (vaccinated) hosts that would otherwise reduce their transmission [81]. Under these conditions, unvaccinated hosts are then subject to more-virulent viruses that cause higher rates of mortality. Such effects have been shown experimentally in Marek’s disease virus of poultry [81], and may also apply to pertussis in humans [82]. These considerations should motivate studies of evolutionary changes in SARS-CoV-2 that could be attributable to selection by imperfect vaccines, especially given the current speed of development for diverse vaccines that will vary in their effects.

## CONCLUSIONS

Evolutionary medicine provides a large suite of empirical and conceptual tools that greatly enhance our ability to study and fight human disease. These tools are especially important when the disease agent evolves, and where human public health interventions impose selection that, intentionally or not, drives evolutionary changes in pathogen traits. Mismatches, conflicts and tradeoffs and evolutionary theory much more broadly, are highly relevant to understanding the SARS-CoV-2 pandemic, and to developing hypotheses for how to study and treat COVID-19. The hypothesis that COVID-19 symptom profile and severity are mediated by interferon-based arms races and mismatches, coupled with hyper-activation of innate immune defenses, is concordant with a diverse set of evidence, and further tests of the hypothesis can lead to novel insights including possible therapies. However, it is important to note that the variability among humans in immune system responses to COVID-19, and in its clinical symptoms, can be accounted for only partially, and in general ways, by predictions of the hypothesis addressed here. Combining hypotheses derived from evolutionary biology with tests and insights from studies of proximate mechanisms and therapies, especially through the use of placebo-controlled prospective clinical trials, should best accelerate progress in understanding and alleviating the pandemic caused by SARS-CoV-2.

## Supplementary data

Supplementary data is available at *EMPH* online.

## Supplementary Material

eoaa036_Supplementary_Data
