# Supplementary material for: Evolutionary medical insights into the SARS-CoV-2 pandemic
Source: Evol Med Public Health. 2020 Oct 14;2020(1):314–22. doi: 10.1093/emph/eoaa036 (PMC7665492; doi:10.1093/emph/eoaa036)
Supplement: eoaa036_Supplementary_Data [file eoaa036_supplementary_data.docx]

Supplementary Material

Phenome-wide association study methods were developed in part for exploratory analysis, and validation, of medical drug targets using the links of specific SNPs with diseases, drugs, and other phenotypes. In this analysis, the seven SNPs associated with Covid-19 mortality by Ellinghaus et al. (2020) (rs657152 and rs11385942) and Lu et al. (2020) (rs150892504, rs138763430, rs117665206, rs147149459, and rs151256885) were tested for phenotypic associations using GWAS Atlas (<https://atlas.ctglab.nl/>) (Watanabe al. 2019), which included GWAS of 600 traits from the UK Biobank.

Supplementary File 1 (xls)

PheWAS results from all seven SNPS, sorted by trait; colors correspond to the seven SNPs as follows:

rs657152 yellow-orange, rs11385942 red, rs150892504 black, rs138763430 blue, rs117665206 green, rs147149459 purple, and rs151256885 grey.

Supplementary File 2 (csv), PheWAS results by SNP designation

PheWAS results for each of the seven SNPs separately, with traits sorted by p-value.
